# Overcoming the translational crisis of contemporary psychiatry – converging phenomenological and spatiotemporal psychopathology

**DOI:** 10.1038/s41380-023-02245-2

**Published:** 2023-09-13

**Authors:** Georg Northoff, Jonas Daub, Dusan Hirjak

**Affiliations:** 1 Mind, Brain Imaging and Neuroethics Research Unit, The Royal’s Institute of Mental Health Research, University of Ottawa, Ottawa, ON Canada; 2 Department of Psychiatry and Psychotherapy, Central Institute of Mental Health, Medical Faculty Mannheim, University of Heidelberg, Mannheim, Germany

**Keywords:** Schizophrenia, Neuroscience

## Abstract

Despite all neurobiological/neurocomputational progress in psychiatric research, recent authors speak about a ‘crisis of contemporary psychiatry’. Some argue that we do not yet know the computational mechanisms underlying the psychopathological symptoms (‘crisis of mechanism’) while others diagnose a neglect of subjectivity, namely first-person experience (‘crisis of subjectivity’). In this perspective, we propose that Phenomenological Psychopathology, due to its focus on first-person experience of space and time, is in an ideal position to address the crisis of subjectivity and, if extended to the brain’s spatiotemporal topographic-dynamic structure as key focus of Spatiotemporal Psychopathology, the crisis of mechanism. We demonstrate how the first-person experiences of space and time differ between schizophrenia, mood disorders and anxiety disorders allowing for their differential-diagnosis – this addresses the crisis of subjectivity. Presupposing space and time as shared features of brain, experience, and symptoms as their “common currency”, the structure of abnormal space and time experience may also serve as template for the structure of the brain’s spatiotemporal neuro-computational mechanisms – this may address the crisis of mechanism. Preliminary scientific evidence in our examples of schizophrenia, bipolar disorder, anxiety disorder, and depression support such clinically relevant spatiotemporal determination of both first-person experience (crisis of subjectivity) and the brain’s neuro-computational structure (crisis of mechanism). In conclusion, converging Phenomenological Psychopathology with Spatiotemporal Psychopathology might help to overcome the translational crisis in psychiatry by delineating more fine-grained neuro computational and -phenomenal mechanisms; this offers novel candidate biomarkers for diagnosis and therapy including both pharmacological and non-pharmacological treatment.

## Introduction

**Fig. 1:**
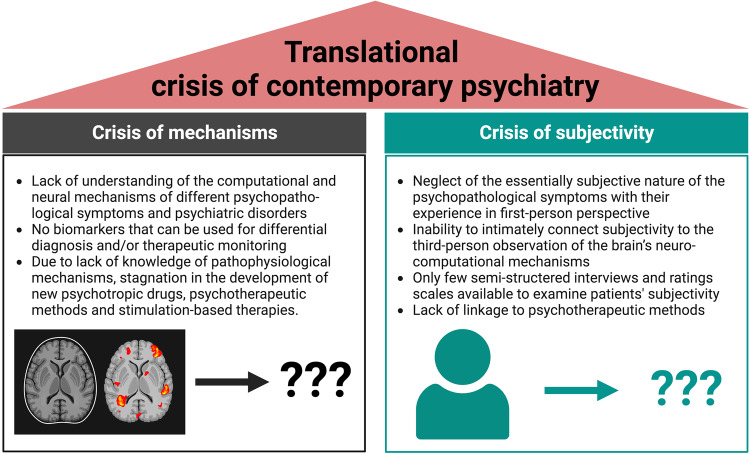
Translational crisis of contemporary psychiatry. This figure illustrates important aspects contributing to the crisis of mechanism and the crisis of subjectivity.

Psychiatry made enormous scientific progress in the last 50 years in various fields including genetics and brain imaging. However, recently some authors declared a ‘crisis of contemporary psychiatry’ [1–3], because both disciplines failed to deliver the promised results for daily clinical application in diagnosis and therapy [4]. While some authors suggest to more strongly develop computational psychiatry to resolve the ‘crisis of mechanisms’ [3, 5], others propose that there is lack of appreciation of the subjective component of psychopathological symptoms [6–11] (Fig. 1). The subjective component is, for instance, taken into focus in Phenomenological Psychopathology (PP) [12, 13] which targets first-person experience of time and space, self, body and world as the subjective core of psychopathological symptoms and/or related cognitive-affective function [14–17]. However, this leaves open how first-person experience including the psychopathological symptoms link to third-person observation about the brain’s neural activity changes.

The main claim of our perspective is to show how to address this gap in our current knowledge. We propose that PP provides an ideal stepping stone to address both the crisis of subjectivity and crisis of mechanism. For that, PP needs to converge with what recently has been introduced as Spatiotemporal Psychopathology (STPP) [18–20]: the key assumption here is that brain, experience and symptoms are intimately connected through their shared basic spatiotemporal disturbance as a “common currency” [18, 21]. Such merger of PP with STPP can address both the crisis of subjectivity and the crisis of mechanisms. This shall be illustrated by space and time experience as well as their possibly related neuronal correlates in schizophrenia (SZ), mood (uni- and bipolar) and anxiety disorders (AD). The two sections on space and time organization in both experience and brain share the same structure: First, we introduce the historical and phenomenological origins of the spatiotemporal concepts as such. Second, we discuss those EEG and MRI studies in SZ, bipolar disorders (BD), major depressive disorders (MDD) and AD that conjointly examined subjective experience of space or time including their neuronal correlates (see supplement for methodological approach). We hypothesize that the altered experience of space and time in psychiatric disorders can be mapped onto correspondingly altered spatiotemporal dynamics of the brain’s neural activity. This might provide the currently elusive link of brain, first-person experience and psychopathological symptoms that makes it possible to develop differential-diagnostic markers for clinical practice.

## Spatial dimension – clinical differential diagnosis and neuro-computational mechanisms

### Subjective experience of space

Among the historical authors who used PP to examine subjective experience of space are Karl Jaspers (1883–1969), Hans Sexauer (unknown), Ludwig Binswanger (1881–1966), Klaus Conrad (1905–1961), and Paul Matussek (1919–2003), among others. For instance, Jaspers described a SZ patient who experienced infinite space [22]; Jaspers talks about *“space with an atmosphere“* [22]. A SZ patient of Sexauer reported: *“If you put the sofa cushions in disorder, everything is blurred - the laws as well. It’s just such a hustle and bustle in the whole world. The points of the compass, as you learned them as a child - north is no longer, as it used to be, the North Pole, the cold. Today, the differences are no longer like that, you can swap them around like a spinning top. I just orient myself to what’s here in the room around me*” [23p. 814]. Binswanger described a patient who, *“lying in bed, sees and feels how a piece of the railroad body, which is located some distance below his window, comes up into his room and penetrates into his head. There is palpitation, fear that the light of life will go out, and a severe headache in the forehead due to the track penetrating into the brain. The sick person is perfectly oriented in the oriented space, he knows that the railroad body lies down there and remains lying; at the same time, however, he sees it coming up and is fully aware of this spatial discrepancy, explicitly declaring that it is ‘something so stupid, so stupid’ that one knows one thing and still experiences the other”* [24p. 634]. Interestingly, the patient experiences a unity with the space surrounding him. The space is divided here into a normal and a pathologically altered space, because the experience of the appearance of the rails and their penetration into his head also takes place in this space.

Conrad [25] described the development of SZ as a gradually progressive process that begins with an essential change and a considerable fragmentation and constriction of the overall psychic field against the background of an indefinite “imminence” or “alienation” [25p. 41]. The space around the patient feels unsafe and threatening. The patient does not know what is going on, he/she is extremely confused. Conrad called this anxious state of tension “trema”. Later on, Matussek described three *Ganzeigenschaften* (English: general characteristics) of perceived objects in the space surrounding the patients, namely the structure, the whole nature or quality (German: *Ganzbeschaffenheit oder –qualität*), and the essence (German: *Wesenseigenschaft*) [26p. 293]. According to Matussek, the essential disturbance in the experience of SZ patients lies in the observation of alternate properties of an object, a situation, or an event in the space surrounding him/her: it is not a change of the considered object or event in and by itself, that is, its basic structure or quality, but its increased and expanded emergence within abnormally experienced spatial context [26p. 287]. Further, a 45 year old male patient treated at Central Institute of Mental Health (CIMH) described his disturbed spatial experience as follows: *“Things merge, it’s like being stuck. Other people were too close to me and threatened me.”* Another 27 year old female SZ patient treated at CIMH stated: *“I felt at that time as if I were in another room, in the basement. This room was set up exactly like the room I was actually in, except that in my imagination it was underground. When I left the room straight ahead, I thought I had climbed a flight of stairs.”* Finally, a 26 year old female patient treated at CIMH reported about her experience of dissolving boundaries between her body and the space surrounding her: *“This foreign feeling is often there, although I know the place or way well. This happens mainly when I feel a sensory overload. I then feel like a ping-pong ball. I feel a physical restlessness and tension and have lost my orientation. […] The body boundaries of the skin dissolve. It’s alternating big and small, pulsating like ray movements or vibrations. […] The walls are getting closer and closer to me - exponentially. It doesn’t come to a bang, though, but feels like it’s right in front of it and feels like I’m being crushed by the walls. When I change position, the feeling subsides. I don’t know exactly how long these experiences last, probably between 10* *min and half an hour. […] I step on a border and enter another area that is not mine and is foreign to me.”*

Together, both the historical and contemporary patient cases illustrate that SZ experience is characterized by an abnormal blurriness and fragmentation of the spatial boundaries between self, body and world. This serves as the basis for many of the psychopathological symptoms such as delusions, ego disturbances and aberrant orientation in the environment. In addition to qualitative phenomenological studies, a recent study by Stanghellini et al. [27] examined the abnormal space experience (ASE) in a large sample of SZ patients. This study showed that the experience of blurring spatial boundaries between self, body and environment are a key feature of the puzzling metamorphosis of the SZ lifeworld. Hence, this study [27] empirically confirmed what Jaspers, Sexauer, Conrad and Matussek described in more qualitative terms. What the above authors all share and described in similar terms is that the spatial separation of the own person from other people or objects is no longer existent in SZ. In particular, SZ patients have the experience of being penetrated/invaded by other people and objects in the personal/peripersonal space around them. There is a dissolution of spatial boundaries between SZ patients, other persons and objects; this can lead to the development of delusions and specifically delusions of alien control as well as of self- or ego-disturbances (passivity phenomena) [28]. More broadly speaking, this reflects the confusion of internally- and externally-oriented cognition as it is typical for SZ [29]. Besides ego-disturbances in terms of passivity phenomena, SZ patients also experience porous borders between self and other persons that lead to difficulties in their social cognition including mindreading or the misinterpretation of facial emotions. Due to the threatening nature of such experiences, SZ patients tend to withdraw socially and avoid any interaction with others. A 33 year old male patient treated at CIMH stated: *“In the acute phase, I retreat into a shell to protect myself as things blur and boundaries are lost.”*

Are these spatial experiences specific for psychosis and SZ? To address this, we turn to spatial experience in mood disorders. On the one hand, MDD patients experience a constriction of their perceived sensorimotor space [30], their movements are slowed, and other people and objects are difficult for them to reach [31, 32]. Fuchs speaks of *“a gap between the body and its surroundings”* [30]. Stanghellini also offers a similar explanation when he claims that patients with melancholia often experience their body *“as an obstacle between the self and the world”* [33]. A 28 year old female patient with MDD treated at CIMH described her disturbed spatial experience similarly: *“It is as if I am sometimes beside myself. The environment then seems colorless and pale and the distance to other people becomes huge, I feel rejected and ignored.”* In MDD, contact with other people and the environment seems to be far away, leading to social isolation. Another MDD patient treated at CIMH described her experience as follows: *“In the first breakdown, I felt like I was heavy as lead and something pushed me into bed. Others seemed far away and I felt isolated and distanced from my surrounding.”*

On the other hand, manic BD patients tend to have the feeling - in the context of delusions of grandiosity - that everything is within their reach [31, 32]. The patient has the impression of having all the possibilities in the world, everything seems to be within reach. One’s own radius of action is enormously extended due to the increased drive, euphoric mood and sleep disturbances (the day seems longer). According to Binswanger *“The world is too small for this being in expansion […] and distances become smaller”* [34]. Fuchs postulated that *“the relation of person and space is characterized by centrifugal dispersion and dedifferentiation“* [30]. In relation to the changed experience of space, manic patients also experience metamorphosis of their lived body. Stanghellini described a manic patient who experienced his body turning upside down and expanding in space: *“Just the way he was, then, with his body slightly leaning forwards and his head out, and constantly on the tips of his toes; all knotted up in a sort of total spasm, his jaw locked into a lockjaw and his face muscles all rigid, a slow and never-ending process of ‘turning upside down’ started up. […] But the biggest relief came from noting how his brain, finally freed into the open air, could fill up a much bigger space that what had been reserved for it ‘right side up’ inside the cranial cavity.”* [33, pp. 139–140]. This case report impressively illustrates the so-called ‘maniacal corporeality’ [33, pp. 139–140]. The lived body gains an increased fluidity, flexibility, and mobility. These are the characteristics that lead to the assumption that these patient have unlimited possibilities in their repertoire of action and behavior.

Taken together, the experience of space clearly differs between SZ and MDD/BD. SZ patients experience fragmentation with the blurring of the spatial boundaries between self, world and body. While MDD and BD patients experience an abnormal constriction or extension of their existing space. Furthermore, the fact that the spatial experience can be disturbed in different ways in SZ and MDD/BD provides evidence that space experience can support differential-diagnostic considerations. Pending further quantitative studies with the development of proper psychometric scales, this renders space experience a strong candidate for clinical differential diagnosis.

### Spatial measures of the brain’s topography and its neuro-computational mechanisms

Unfortunately, we could not identify a single MRI study that investigated the neural mechanisms of aberrant spatial experience in psychiatric disorders. Therefore, we concentrated on MRI studies that focused on the spatial changes/features of brain networks dynamics in SZ. But what does a spatial change in network dynamics exactly mean? Recent analytical approaches have made it possible to measure rapid shifts in activity across different networks (see also [35, 36]). A recent study by Wang et al. [37] examined the changes in dynamic brain states present in SZ patients and found that three states - co-activated brain areas (e.g. different activation patterns of fronto-parietal control network, sensorimotor areas, visual cortex, insula and default network) - occurred less often in SZ patients than in healthy controls (HC), even though the spatial maps of these states appeared to be similar between the two groups [37]; this suggests specific dynamic changes in the otherwise similar network topography. Another MRI study by Iraji et al. [38] examined spatial dynamics within and between brain networks in SZ. The authors concluded that the brain reorganizes its various networks at different spatial scales including shorter and longer ones; this is expressed at the macro level in dynamic changes in the variations of the spatial coupling among networks and their functional domains (cognition, affect, sensorimotor, etc.). Interestingly, a very recent resting-state fMRI study by Pan et al. [39] examined the dynamic reconfiguration of the brain, i.e., the dynamic spatial interactions/changes between particular brain regions for diagnostic purposes. This study proposed a spatiotemporal dynamic functional connectivity method for the diagnosis of SZ [39], i.e., obtaining a significantly higher classification accuracy (81.82%) than other computational methods. A similar resting-state fMRI study by Kottaram et al. [40] corroborated these findings and showed that the combination of both spatial and temporal dynamics of functional connectivity is able to predict diagnostic status with high accuracy exceeding 90%. Together, these findings suggest dynamic changes in the network topography of SZ.

Going beyond functional networks by taking a global approach to the brain, Yang et al. [41] investigated how fMRI global signal activity is represented in single regions and networks, e.g., global signal topography (see also Zhang and Northoff [42] for review). SZ patients showed a converse pattern of global signal topography in sensorimotor regions (low in SZ) and higher-order associative (high in SZ) regions compared to HC. Given that sensorimotor and higher-order regions are associated with externally- and internally-oriented cognition respectively [43–45], the reversal in their global signal topography in SZ may contribute to these patients’ experience of blurring spatial boundaries between internal self and external world. Correspondingly, a review/meta-analysis [29] demonstrated that SZ patients show decreased neural changes (mainly EEG) during the transition from the internal prestimulus activity to external task-related activity – that entails a lack of distinction of the external task from the ongoing internal cognitions. What is experienced as the blurring of spatial boundaries between the internal self and the external world may thus be mediated by corresponding confusion of internal (prestimulus, higher order regions) and external (task-related, sensorimotor) activity on the neural level of the brain’s intrinsic topography and its dynamics (that is, its changes over time). Accordingly, the spatial-topographic (and temporal-dynamic) confusion of internal and external activity/events may be shared by both experiential and neural levels as their “common currency” in SZ.

**Fig. 2:**
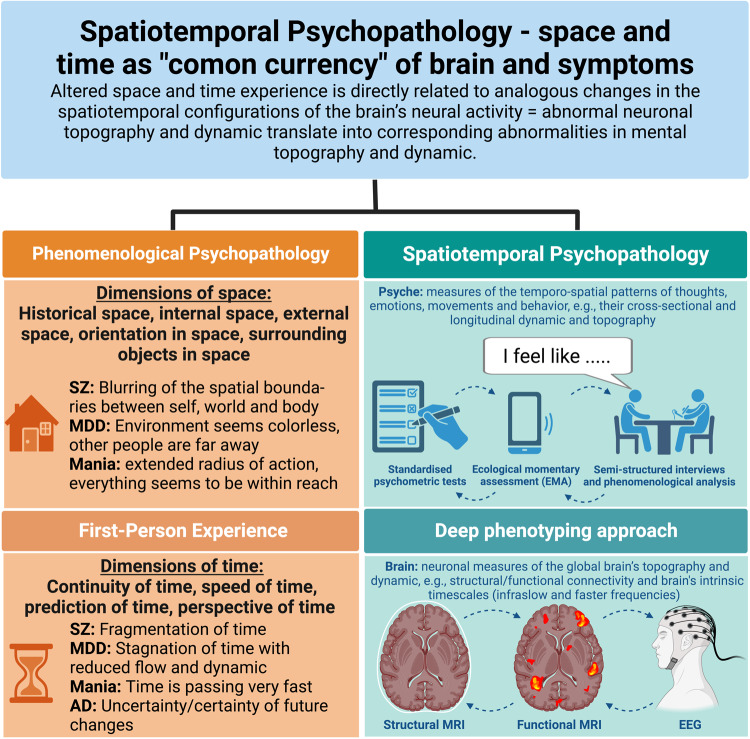
Spatiotemporal Psychopathology - space and time as “common currency” of brain and symptoms. This figure illustrates the main concepts and methodological approaches of phenomenological and spatiotemporal psychopathology leading to a common currency of altered space and time experience. Both dimensions can be studied through the examination of the first-person experience and brain networks (=deep phenotyping approach).

Different topographic changes are observed in MDD [46, 47] and BD [48]. Here, the brain’s topographic organization is shifted towards its inside, that is, towards its default-mode network at the expense of the sensorimotor regions in depressed states of MDD and BD while the opposite can be observed in manic states of BD [46–48]. Does such an inside/inwards constriction or outside/outwards extension of the brain’s topography correspond to the subjects’ experience of a restricted (depression) or extended (mania) subjective space? Future studies combing neural and phenomenological measure are warranted to support such hypothesis. These will also show whether the currently observed differences in brain topography including their relationship to the experience of space can serve as candidate biomarkers for the differential-diagnosis of SZ vs MDD vs BD (Fig. 2).

## Temporal dimension – clinical differential diagnosis and neuro-computational mechanisms

### Subjective experience of time

The examination of anomalous time (and space) experience in psychiatry was initiated in the 1920s by Eugène Minkowski (1885–1972), Ludwig Binswanger and Erwin Straus (1891–1975), among others. In particular, both Minkowski and Binswanger postulated aberrant time-space experience as a *“basic disturbance”* ( = *“trouble generateur”* coined by Eugène Minkowski) in psychiatric disorders. According to Straus [49, 50], the time experience is a medium of subjective experience in general. This said, all experiences, thoughts, actions and emotions/affects of psychiatric patients are dependent on changes in the temporal experience in terms of form and content (for overview see also [51]). More recently, Stanghellini et al. [52, 53] conducted a semi-quantitative investigation about the subjective experience of time in SZ and MDD. This study showed temporal fragmentation in SZ featured by disruptions in time flow, déjà vu, and premonitions about the self and the world [52]. Relying on earlier [54] and current [55, 56] phenomenological studies, the authors assumed a basic disturbance in the articulation or synthesis of time in SZ.

A 26 year old female SZ patient treated at CIMH described her fragmented time experience as follows: *“In my first acute phase, I was only concerned with the current delusions. The past and the future did not play a role, there is then only the current urge. I also had the impression that several things were happening at the same time. Further, I felt daily cracks in time that I did not experience as continuous. […] I experienced time as if it were in individual blocks, some lasting only a few minutes and running like a program. Getting up, brushing my teeth, and having a cigarette in the morning all belong together. After that, there’s a break. Then making the bed, coffee and getting dressed. Sometimes there’s a crack from one minute to the next.”* In SZ, time does not flow properly, but is fragmented into individual scenes, like in a cartoon film with various snapshots not being connected at all. Importantly, these experiences are specific for SZ as they are not reported by MDD patients. Instead, MDD patients rather experience stagnation of time with reduced flow and dynamic [53, 55]. A 53 year old female MDD patient treated at CIMH stated: *“You are stuck in the past and almost petrified, brooding how it could come so far with no perspective for the future. It is associated with feelings of guilt and time passes very slowly for you.”* This case shows that the experience of stagnating time is rather characteristic for MDD patients as it is not observed in SZ. This amounts to a basic disturbance in the conation or inhibition of time in MDD ( = the dynamic of time [19, 57]) as distinguished from the altered construction or synthesis ( = fragmentation) of time in SZ.

How about the distinction between MDD and depressed BD? A recent study investigated subjects’ experience of the timing of their thoughts, e.g., thought dynamic in MDD and BD by letting subjects report the changes in their internally- and externally-oriented thoughts [58]. This study demonstrates that internally-oriented thoughts lasted longer, exhibited slower frequency, and showed less power (as calculated by power spectrum of the time series of their thought content changes) in MDD compared to HC. Importantly, this pattern was similar (long duration of internally-oriented thoughts) and distinct (normal power of thought) in depressed BD patients [58] – this again indicates the differential-diagnostic relevance of the experience of time now with regard to the changes of one’s thought contents over time, that is, thought dynamics. The differential-diagnostic relevance of time experience for SZ as distinct from MDD and BD is further supported by the STEP [59]. Importantly, particular STEP items like the experience of temporal fragmentation and premonitions are indeed specific for SZ as distinguished from both a HC and an affective group [59].

Together, these studies show the feasibility of the time experience as a differential-diagnostic marker for SZ vs MDD vs BD. Importantly, this goes along with the distinction of different dimensions of time like continuity, speed/duration, prediction, and perspective [14, 55, 60]. Continuity of time refers to the experience of flow of time which is disrupted in SZ [56]. Time speed refers to the experience of a certain duration which is often overestimated in AD patients [61] while it is underestimated and thus abnormally slowed down in MDD [14, 62]. Time prediction refers to the experience of uncertainty/certainty of future changes and their time points as it is typically disturbed in AD patients [14, 61]. Finally, time perspective refers to the experience of the relationship of past, present and future which often is abnormal in MDD (experience of strong past) and mania (experience of predominance of future) [14, 55, 60, 63].

### Temporal brain measures and neuro-computational mechanisms

How can the features of the subjective experience of time serve as a template for investigating the brain’s neural and computational mechanisms? The brain exhibits spontaneous activity (as distinct from task-evoked activity) which constructs its own inner time as distinguished from the outer time of the environment [21, 64]. As postulated in STPP, the brain’s spontaneous activity constructs its own ongoing inner time with temporal features that are more or less analogous to the ones that characterize the experience of time, namely continuity/flow, speed/duration, perspective and prediction. Given that analogous temporal features are shared by both brain and experience as their “common currency” [18], the different features or patterns of the experience of time may serve as an ideal starting point or stepping stone for searching the neuro-computational mechanisms in the brain’s spontaneous activity with analogous temporal features. Specifically, the experience of the continuity of time and its temporal flow are closely related to the timing of both phase and amplitude in the neural activity as they can be measured and distinguished in EEG. SZ patients show temporal imprecision in the millisecond range in both phase [65] and amplitude [66] in their neural activity as measured with EEG. Especially the neural findings of temporal imprecision in phase synchrony or entrainment to external stimuli [65, 67, 68] are more or less specific to SZ as distinct from MDD and BD [65, 69]. On the psychological level this is further supported by analogous temporal imprecision in the millisecond range in temporal behavior [60, 62] and time estimation [70, 71]. Finally, these neural, behavioral, and psychological findings showing temporal imprecision are well in line with the experience of temporal fragmentation and premotions in the SZ patients as reported above: these two experiential features may result from an analogous disruption of the continuity of time and its temporal flow on the neural/computational level as based on its temporal imprecision.

Yet another dimension of time is speed. The data clearly show that the MDD subjects experience time as too slow and stagnant (see above) which is manifest in their emotion, thoughts, perceptions, and movements. This is complemented by an analogous speed disturbance on the neural level. The global spontaneous activity is too slow in MDD (and also depressed BD) shifting its power more towards the slower end of the infraslow power spectrum (0.01 to 0.1 Hz) as measured with fMRI [48, 72, 73]. That goes along with decreased neural sensitivity to especially fast negative stimuli in both motor cortex and DMN whose reduced stimulus-evoked activity correlates with the degree of psychomotor retardation [74]. Accordingly, instead of temporal imprecision with disruption of temporal continuity as in SZ, MDD can rather be characterized by a disturbance of time speed as these patients are too slow in both their neural and experiential and cognitive-behavioral activity.

Finally, time prediction is yet another dimension of time. This concerns how a previous time point can predict the possible changes and events at a future time point which is closely related to the experience of certainty/uncertainty. AD patients typically suffer from the experience of uncertainty [14, 61]. On the neural side, one can observe desynchronization among different regions (showing decreased functional connectivity) especially in the CMS and default-mode network during both rest and task states [75, 76]. If the regions are not properly synchronized with each other, one can no longer temporally predict from one region’s neural activity to another which, on the computational level, has been described as uncertainty [61]. However, future studies are necessary to connect such lack of temporal prediction on the neural level with both computational uncertainty and the experience of temporal uncertainty featured by the inability to predict future events in AD patients.

Taken together, we can see how the different dimensions of the experience of time including their disorder-specific changes in SZ, MDD, BD and AD are accompanied by more or less corresponding neural and computational changes in the temporal features of the brain’s neural activity (Fig. 2). This provides the first steps towards what has been described as “Computational phenomenology” [77] or, as we would extend, “Spatiotemporal computational phenomenology”. Specifically, the experience of time can serve as a template to guide neuro-computational investigation – this allows extending PP beyond experience to the brain. One can link those first-person experiential features of time that allow for clinical differential-diagnosis of SZ, MDD, BD and AD (see above) to specific third-person observable measures of temporal dynamics in the neuro-computational mechanisms of the brain (and also the simulated models). While clinically, we can then use these experiential markers of time in conjunction with more or less corresponding neuro-computational markers of time, e.g., dynamics in the differential-diagnosis of SZ, MDD, BD and AD.

## Conclusions and recommendations

**Fig. 3:**
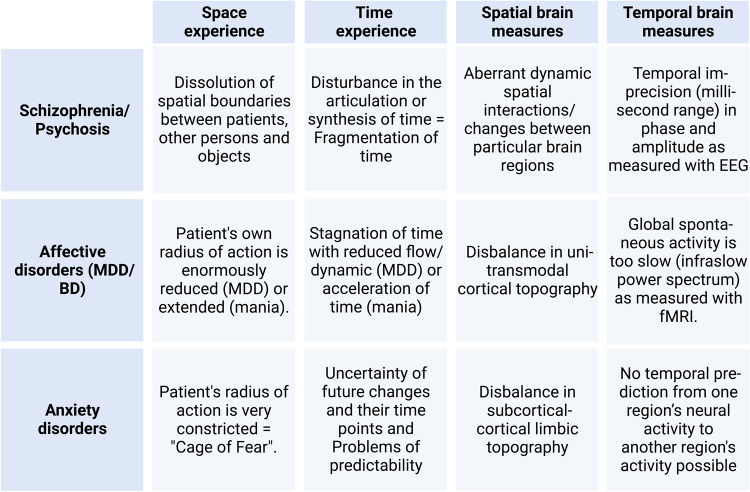
Spatiotemporal criteria in subjective experience and brain measures for differential-diagnosis of schizophrenia/psychosis, major depressive (MDD), bipolar (BD) and anxiety disorders. This figure highlights the relevant criteria of spatiotemporal phenomena specified for both the first-person experience of space and time and the spatiotemporal brain measures organized along the lines of psychiatric diagnoses.

PP has a long history of identifying disturbances in space and time experience which is currently further developed in SZ and MDD [13]. The concept of the basic disturbance implies that we need to look for common spatiotemporal features that are fundamental to and manifest across the various symptom domains, e.g., motor, sensory, affective, cognitive, and social. As pointed out in our examples, different disorders like SZ, MDD, BD and AD display different kinds of basic spatiotemporal disturbances in both their subjective experience and neural activity; this, as we suggest, can be used in clinical differential diagnosis (Fig. 3).

Use of spatiotemporal experience in clinical diagnosis requires quantitative measures and tools. Focusing on mainly individual qualitative reports so far, PP needs to specify the subjective experience of space and time in a more granular and quantitative way. For instance, we may want to ask patients in our clinical interviews to self-report and assess their experience of time (and space) in the depressed state or when they are manic. Going beyond qualitative (or semi-qualitative) accounts, this requires the use of systematic-quantitative clinical scales (and ideally also behavioral tests) for the differential experience of space and time in SZ, MDD, BD and AD. There are already several validated instruments in the English and German language, which fully or at least partially assess the altered spatial and temporal experience of patients with psychiatric disorders. These include the semi-structured qualitative and semi-quantitative psychometric interview EASE (Examination of Anomalous Self-Experience) [78], the semi-structured interview EAWE (Examination of Anomalous World-Experience) [79, 80], and the STEP scale (Scale for Space and Time Experience in Psychosis) [59, 81]. Interestingly, STEP confirmed the experience of blurriness and fragmentation of spatial boundaries in SZ [59] and was able to show differences between spatial experience of SZ and MDD patients [59]. That might contribute to resolving the crisis of subjectivity by rendering its investigation more scientific, e.g., quantitative, without losing the subjective-experiential core of psychopathological symptoms.

Further, PP needs to connect first-person experience of space and time to more or less analogous spatiotemporal features in the brain’s neural activity, e.g., its topography and dynamics as observed in the third-person. We assume that altered space and time experience (as the most bottom layer of mental disorders signifying their basic disturbance) [82] is directly related to analogous changes in the spatiotemporal configurations of the brain’s neural activity – abnormal neuronal topography and dynamics translate into corresponding abnormalities in mental topography and dynamics [20]. The neural and computational mechanisms driving the neural activity changes including their manifestation in the various psychopathological symptoms are then identified as spatiotemporal mechanisms. In the future, a combination of different methods (e.g., phenomenological, psychological/STEP-based and neurobiological/MRI-topography and dynamics based) might allow to distinguish patients with SZ from those with MDD, BD and AD on the basis of their basic spatiotemporal disturbance in both brain and subjective experience – this contributes resolving the crisis of mechanisms.

In sum, we are encountering a translational crisis of contemporary psychiatry as both neurobiological/neurocomputational and phenomenological approaches to mental disorders seem to resist translation into clinical practice. We propose that PP provides an ideal starting point and template to resolve the crisis of psychiatry when converging its investigation of spatiotemporal experience with the spatiotemporal, e.g., topographic and dynamic investigation of the brain as proposed in STPP which allows translating basic research into clinical practice.
